# A cross-sectional study on the influence of emotion regulation strategies on the association between trait mindfulness and suicidality

**DOI:** 10.1186/s40359-025-02530-5

**Published:** 2025-04-05

**Authors:** Inken Höller, Judith Martens, Christina Fönschau, Thomas Forkmann

**Affiliations:** 1 Department of Clinical Psychology and Psychotherapy, University of Duisburg-Essen, Universitätsstraße 2, 45141 Essen, Germany; 2 Department of Clinical Psychology and Psychotherapy, Charlotte Fresenius Hochschule, Düsseldorf, Germany

**Keywords:** Mindfulness, Emotion regulation, Suicidality

## Abstract

**Background:**

Several risk factors for suicidality, including dysfunctional emotion regulation strategies (ERS), have been identified. With regard to the high number of suicides worldwide, suicide prevention and identifying potentially protective factors is of high relevance. Mindfulness has been discussed to positively influence both suicidality and ERS (e.g., expressive suppression, thought suppression, and cognitive reappraisal). The aim of this study was to examine associations between mindfulness, ERS, and suicidal ideation as well as the mediating role of ERS on the association between mindfulness and suicidal ideation.

**Methods:**

In a cross-sectional online study, 376 participants (*M* = 27.35, *SD* = 9.98, range = 18–77; *n* = 282 (75%) female) filled out questionnaires on mindfulness (Freiburger Questionnaire on Mindfulness; FFA), suicidal ideation (Beck Scale for Suicidal Ideation; BSS), expressive suppression (Emotion Regulation Questionnaire; ERQ), thought suppression (White Bear Suppression Inventory; WBSI), and cognitive reappraisal (ERQ). Correlations and mediation models were calculated using SPSS.

**Results:**

Mindfulness was negatively correlated with the use of expressive suppression and thought suppression as well as positively correlated with cognitive reappraisal. Suicidal ideation was negatively correlated with mindfulness and cognitive reappraisal and positively with expressive suppression and thought suppression. The mediation models showed that all three ERS mediated the relationship between mindfulness and suicidal ideation.

**Conclusions:**

The results meaningfully complement other findings in the field and show how promising it can be to integrate mindfulness-based interventions in suicide prevention. Additionally, ERS can be a starting point for therapeutic interventions.

**Trial registration:**

The study was preregistered on 05/06/2022 at aspredicted.org (#96242) prior to any data collection (see https://aspredicted.org/a4yq7.pdf).

## Background

Emotion regulation strategies (ERS) are necessary for individuals to influence the intensity of their emotions and to modify their emotional expression and experience. Emotions are a multi-facetted phenomenon, which influences subjective sensations on a psychological, physiological, and behavioral level [1]. From a psychological perspective, emotions can change the focus of attention and, thereby, influence a sequence of actions and the intensity of memories. From a physiological perspective, emotions lead to an activation of the autonomous nervous system [2]. From a behavioral perspective, emotions are not only feelings but at the same time a motivation for action including mimicry, gestures, change in body posture, or situation-related action [3]. Depending on the situation, emotions can be useful or not. As a consequence, in some situations a regulation of emotions and their consequences is required.

ERS can be either antecedent-focused or response-focused [4, 5]. Antecedent-focused regulation strategies start after having received a stimulus, but before forming the actual emotion. In contrast, reaction-focused strategies are usually applied after forming the emotion. Those strategies change only the expressive component of an emotion [6]. Within these two broad classes of emotion regulation, there is a distinction between five different categories of strategies. Four of these strategies belong to antecendent focused emotion regulation, namely situation selection (situations and people are chosen or avoided based on their emotional impact), situation modification (the situation is altered to change its emotional impact), attention deployment (directing attention towards or away from something in order to regulate one´s emotions), and cognitive change (the situation one is in, or the ability to cope with it, is reappraised to alter emotions) [6]. One form of cognitive change is cognitive reappraisal. Using cognitive reappraisal results in the reduction of the experiential and behavioral components [7]. The emotion evoking stimulus will be neutralized because its meaning will be re-evaluated [5].

The fifth category, called emotion modulation, belongs to the response-focused emotion regulation and involves strategies used after the full generation of an emotional response [6]. Important examples are expressive suppression or thought suppression [5]. While expressive suppression starts at the behavioral component (f. ex. the oppression of crying in a social situation if it is not wanted for others to see) [7], thought suppression can already influence the outcome of the experienced emotion by consciously drawing the attention from one thought to another. Evidence suggests that this can lead to paradox effects, because the oppression of a thought can lead to an unconscious process, that urges this thought back to consciousness [8]. In the long run, the frequency and intensity of this thought increases and leads to a rebound effect [8]. Thus, suppression only reduces the expression, but not the experience of negative emotions, these emotions can persist and accumulate. Additionally, suppression requires substantial energy and cognitive resources [7]. Due to the named mechanisms of the different strategies, cognitive reappraisal can be seen as a functional strategy that leads to psychological well-being [7]. In contrast, expressive suppression and thought suppression have been associated with multiple psychological malfunctioning [7, 8] and psychopathology, such as mood and anxiety disorders [9]. There has been evidence for robust associations between expressive suppression as well as thought suppression and the frequency of occurrence of suicidal ideation [8, 10, 11]. Pettit et al. [8] showed that participants, who frequently oppressed thoughts reported suicidal ideation more often (even when statistically controlling for depression). Forkmann et al. [10] revealed in a sample of inpatients with a depressive disorder that those with suicidal ideation reported more expressive suppression and less cognitive reappraisal than inpatients without suicidal ideation. A recent study by Franz et al. [11] investigated the moderating effect of ERS on the association between stress and suicidal ideation. They found a main effect of stress on suicidal ideation in participants using mostly expressive suppression as ERS. This effect was not found for patients using cognitive reappraisal.

One protective factor for suicidality, that has come to the fore in both therapy and research, is mindfulness [12]. In a case study of Williams et al. [13], mindfulness based cognitive therapy (MBCT) was investigated in a patient suffering from suicidal ideation and a past suicide attempt. After eight weeks of therapy, results suggest that mindfulness provides tools that help to regulate emotions and to develop different approaches to cope with negative emotions [13].

Even though, mindfulness has suffered criticism for not being consistently, clearly, and precisely defined [14], there has been consensus on certain components included in mindfulness, such as attention steering, emotion regulation and change of perspective [14]. It is therefore not surprising that there has been evidence for mindfulness influencing the choice of ERS (more cognitive reappraisal, less expressive and thought suppression) and thereby the frequency of suicidal ideation [15–18]. Mindfulness leads to positive affect and good psychological well-being [16]. Further mechanisms through which higher mindfulness leads to more psychological well-being may be mindful awareness, reperceiving, exposure, acceptance, attentional control, memory, values clarification, and behavioral self-regulation [19]. This is in line with findings on mindfulness training showing that intense mediation training can help manage the brain’s limited resources [20, 21]. When we pay attention to something, the brain areas related to that thing become more active, while the areas related to things we are not focusing on become less active [22, 23]. Brain activity needs oxygen and glucose, which are carried by the blood. Because of this, the brain has limited resources, and it is thought that attention controls how these resources are used [24]. This might explain emotion regulation differences in persons who are high versus low in mindfulness. Mindfulness appears to change or control how we currently allocate our attention to the sensory inputs we need to focus on [24].

Regarding consistent definitions of mindfulness, it is also important to differentiate between state and trait mindfulness. An experimental study of Garland et al. [18] on mindfulness training suggested that state mindfulness does not influence the choice of ERS but trait mindfulness does [18]. Therefore, evidence suggests a temporal connection between mindfulness attitude and the choice of ERS [16, 17]. Even though, mindfulness, ERS and suicidality have been extensively studied, there is no study so far investigating the associations among all three constructs. Additionally, most studies examining mindfulness concentrated on state mindfulness, while information is missing on the influence of general trait mindfulness on both ERS and suicidal ideation.

Therefore, the first aim of this study was to examine correlations between trait mindfulness and the use of ERS, between trait mindfulness and suicidal ideation as well as suicidal ideation and ERS.

### H1

We hypothesized negative correlations between 1a) trait mindfulness and expressive suppression as well as between 1b) trait mindfulness and thought suppression. We hypothesized 1c) a positive correlation between trait mindfulness and cognitive reappraisal.

### H2

Additionally, we hypothesized a negative correlation between trait mindfulness and suicidal ideation.

### H3

We hypothesized positive correlations between 3a) suicidal ideation and thought suppression as well as between 3b) suicidal ideation and expressive suppression. We hypothesized 3c) a negative correlation between suicidal ideation and cognitive reappraisal.

Building on the findings of Brockmann et al. and Hepburn et al. [16, 17], the second aim was to examine whether the three ERS mediate the association between trait mindfulness and suicidal ideation.

### H4

We hypothesized that all three ERS would show significant mediation effects.

We hope that the findings allow to draw conclusions for practical implications.

## Methods

### Sample

In total, 388 participants filled out an online questionnaire. Due to missing data, *n* = 12 participants were excluded, so that *N* = 376 were included in the statistical analyses. Of those participants, *n* = 282 were female (75%). The age ranged between 18 and 77 years (*M* = 27.35, *SD* = 9.98). 335 participants (86.3%) were not married, 40 (10.3%) were married, 11 (2.8%) were divorced, and 2 (0.5%) were widowed.

Eighty-three (22.1%) participants reported to currently suffer from a mental disorder and 50 participants (13.3%) reported to currently undergo current psychological treatment, *n* = 121 (32.2%) reported a mental disorder as well as psychological treatment in the past. Of the 83 people who reported to currently suffer from a mental disorder, 44 (53.01%) stated depression as their first self-report diagnosis, 21 (25.30%) an anxiety disorder, 4 (4.82%) ADHD, 4 (4.82%) PTSD, 2 (2.41%) eating disorders, 3 (3.61%) personality disorders and 5 (6.02%) other disorders.

Twenty-nine (7.7%) participants reported a suicide attempt in the past. Seventy-five (19.9%) participants reported current suicidal ideation.

### Procedure

Participants were recruited between May (right after receiving the Ethic Committee approval) and July 2022 on social networks and announcements at the university of Duisburg-Essen. In the study, convenience sampling was chosen as the sampling method. Therefore, everyone who was interested in the study and fulfilled the inclusion criteria could participate. The study was conducted in a cross-sectional design and as an online study. The processing time was *M* = 24.96 min (*SD* = 9.51). Before starting the study, on the first page of the online questionnaire participants were informed about the purpose of the study, the voluntary nature of participation, data storage and security, and gave informed consent prior to participating by confirming a consent question after the study information at the end of the page. Inclusion criteria were an age above 18 years and sufficient knowledge of the German language. Participants had the opportunity to take part in a raffle with three coupons with the value of 15 € or to gain course credits. The study was positively approved by the Ethic Committee of the University of Duisburg-Essen on the 19th of May 2022 and was in accordance with the Declaration of Helsinki [25]. Additionally, the study was preregistered at aspredicted.org (AsPredicted#96242) prior to any data collection (see https://aspredicted.org/a4yq7.pdf).

### Measures

In the following, only measures relevant for this study are reported.

To assess the ERS expressive suppression and cognitive reappraisal, the Emotion Regulation Questionnaire (ERQ; original: 7, German version: [26]) was used. The ERQ consists of two subscales with four items assessing expressive suppression (e.g. "I control my emotions by not expressing them.") and six items assessing cognitive reappraisal (e.g. "When I want to feel more positive emotion (such as joy or amusement), I change what I’m thinking about"). All items have to be answered on a seven-point Likert scale with different item phrasing. Higher scores indicate higher use of this strategy. The questionnaire has shown good validity and reliability [26]. Internal consistency in the present sample was acceptable for the subscale expressive suppression with Cronbach’s α = 0.79 and good for the subscale cognitive reappraisal with α = 0.87.

To assess the ERS thought suppression, the White Bear Suppression Inventory (WBSI; original: [27], German version: [28]) was used. The WBSI consists of 15 items (e.g. "There are things I prefer not to think about." or "I wish I could stop thinking.") that have to be answered on a five-point Likert scale with different item phrasings. Higher scores indicate a higher use of thought suppression. The questionnaire has shown good validity and reliability [28]. The total scale showed excellent internal consistency with Cronbach’s α = 0.92 in the present sample. A sum score of the WBSI was used to assess thought suppression in general.

To assess trait mindfulness, the Freiburger Questionnaire on Mindfulness (FFA; [29]) was used. The FFA consists of 14 items assessing trait mindfulness on a four-point Likert scale with higher scores indicating higher mindfulness (e.g. "I am open to the experience of the present moment or I pay attention to what´s behind my actions"). A sum score was built to assess general trait mindfulness. Internal consistency was good for the whole scale with Cronbach’s α = 0.87.

To assess suicidal ideation, the Beck Scale for Suicidal Ideation (BSS; original: [30], German version: [31]), a 21-item self-report measure assessing suicidal ideation (SI) on a three-point Likert scale from 0 to 2 with differing statement groups was used (e.g. wish to live: 0 = I have a moderate to strong desire to live; 1 = I have a weak desire to live; 2 = I have no desire to live). The first 19 items were summed up in case items 4 and 5 have been answered at least with 1. Otherwise, patients had a sum score of zero. Higher scores indicate higher suicidal ideation [32, 33]. Internal consistency in this sample was excellent with Cronbach’s α = 0.91.

### Statistical analysis

All statistical analyses were conducted with SPSS version 27.0. The sample was sufficiently powered for the conducted analyses (correlations: 1-β = 0.8, *p* =.05, *R*^2^ = 0.02, *N* = 150; mediation analyses: 1-β = 0.8, *p* =.05, *R*^2^ = 0.02, *N* = 311) [34].

For the first aim of this study– to investigate whether trait mindfulness is correlated with ERS as well as suicidal ideation– Pearson product-moment correlations were calculated (hypotheses 1a)-c); 2, 3a)-c). Correlations ≥ 0.30 were considered as moderate and correlations ≥ 0.50 were considered as strong correlations [35].

**Fig. 1:**
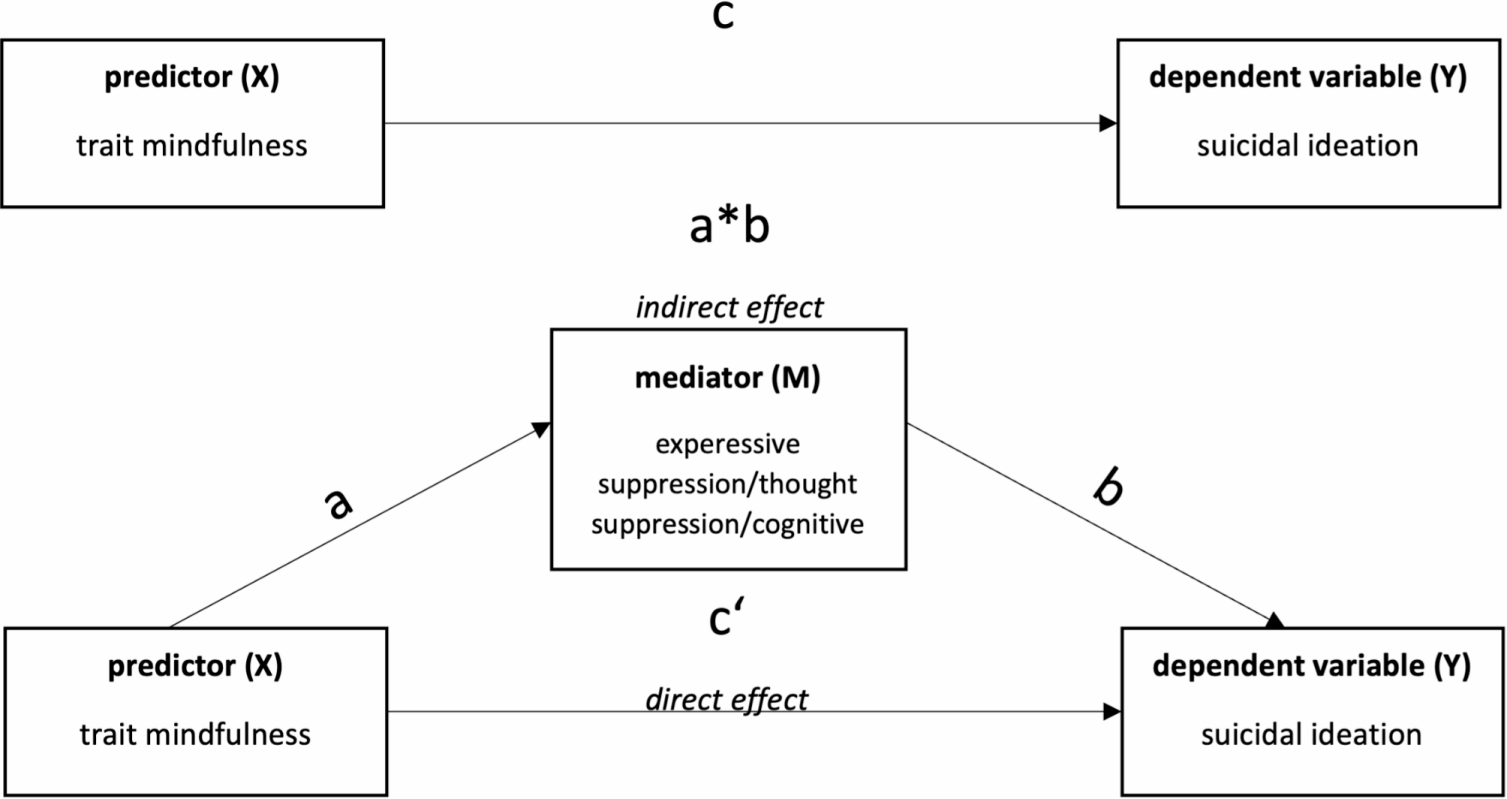
Overview of the calculated effects within the mediation models

For the second aim of this study and to answer the fourth hypothesis whether ERS mediate the relation between trait mindfulness and suicidal ideation– three mediation models were conducted. Mediation model 1 included trait mindfulness as the predictor (X), expressive suppression as the mediator (M) and suicidal ideation as the dependent variable (Y). The second model was the same except that this time thought suppression served as the mediator and the third model included cognitive reappraisal as a mediator. The total effect (the sum of all direct and indirect effects (c)) and the indirect effect, which is the product of the effect of X on M (a) as well as of M on Y (b) were examined [36]. The effect from X on Y in consideration of M (c‘) was calculated. Additionally, we examined whether the indirect effect (ab) became significant [37] (see Fig. 1). For all analyses, 5,000 bootstrap iterations were selected. For all effects, completely standardized effect sizes (CS), which express the respective effects in units of standard deviation, were additionally calculated. Higher values correspond to larger effects. The determination coefficient *R*^*2*^ was also calculated. *R*^*2*^ expresses, how much of the variance of the dependent variable is due to the independent variable [38]. Following Cohen [35], *R*^2^ = 0.02 is considered a weak, *R*^*2*^ = 0.13 a moderate, and *R*^*2*^ = 0.26 a large effect.

## Results

Descriptive information on the measures can be found in Table 1.

**Table 1:** Descriptive statistics of all measures

	*N*	*M*	*SD*	Minimum	Maximum
BSS	376	24.44	6.76	5	52
WBSI	376	50.19	13.34	15	75
FFA	376	36.13	7.01	18	55
ERQ Cognitive Reappraisal	376	27.25	7.57	6	42
ERQ Expressive Suppression	376	14.84	5.29	4	26

Note. BSS = Beck Scale for Suicidal Ideation; ERQ = Emotion Regulation Questionnaire; FFA = Freiburger Questionnaire on Mindfulness; WBSI = White Bear Suppression Inventory

### Correlations

As expected and in line with hypotheses 1a)-b) and 2), we found small to moderate negative correlations for mindfulness and expressive suppression, for mindfulness and thought suppression, and for mindfulness and suicidal ideation. In line with hypotheses 3a)-b) suicidal ideation was moderately positively correlated with expressive suppression and thought suppression. We found a strong positive correlation for mindfulness and cognitive reappraisal (hypotheses 1c) as well as a moderate negative correlation between suicidal ideation and cognitive reappraisal (hypotheses 3c) (for more details see Table 2).

**Table 2:** Correlations of mindfulness. Emotion regulation strategies, and suicidal ideation

	FFA	ERQ Expressive Suppression	ERQ Cognitive Reappraisal	WBSI	BSS
FFA					
ERQ Expressive Suppression	− 0.257 ^**^				
ERQ Cognitive Reappraisal	0.528 ^**^	− 0.220 ^**^			
WBSI	− 0.419 ^**^	0.358 ^**^	− 0.227^**^		
BSS	− 0.345 ^**^	0.252^**^	− 0.301 ^**^	0.337 ^**^	

Note. All correlations were significant (*p* <.01). ERQ = Emotion Regulation Questionnaire; FFA = Freiburger Questionnaire on Mindfulness; WBSI = White Bear Suppression Inventory, BSS = Beck Scale for Suicidal Ideation

### Mediation models

Regarding the mediation analyses, all coefficients were significant (*p* <.001). The total effect of mindfulness on suicidal ideation was reduced when taking into account the mediators (expressive suppression: *c* = − 0.333, *SE* = 0.058; *c‘* = − 0.291, *SE* = 0.059; thought suppression: *c* = − 0.333, *SE* = 0.058; *c‘* = − 0.239, *SE* = 0.061; cognitive reappraisal: *c* = − 0.333, *SE* = 0.058; *c‘* = − 0.250, *SE* = 0.067).

There was an indirect effect from mindfulness on suicidal ideation, which was mediated by expressive suppression (*ab* = − 0.044, *SE* = 0.059, 95%-CI [-0.1465, − 0.0284], see Table 3). The completely standardized indirect effect was *CS*_ab_ = − 0.300. Following Cohen [35], all determination coefficients indicate a medium amount of explained variance (*R*^2^ > 0.11) as well as a significant model (*p* <.001). Mindfulness explained only a small proportion of variance in expressive Suppression (6.6%); correspondingly, the effect was small *a* = − 0.194 (*SE* = 0.024). Still the explained variance for suicidal ideation increased from 11.9 to 14.7% when expressive suppression was considered as a mediator in this model.

There was also an indirect effect from mindfulness on suicidal ideation, which was mediated by thought suppression (*ab* = − 0.094, *SE* = 0.023, 95%-CI [-0.1442, − 0.0508]). The completely standardized indirect effect was *CS*_ab_ = − 0.098. Mindfulness explained a larger portion of variance in thought suppression (17.5%) than in expressive suppression (6.6%). Additionally, explained variance for suicidal ideation increased from 11.9 to 16.4%, when thought suppression was considered as a mediator in the model.

In the last mediation model, there was also an indirect effect from mindfulness on suicidal ideation, which was mediated by cognitive reappraisal (*ab* = − 0.084, *SE* = 0.030, 95%-CI [-0.1465, − 0.0284]). The completely standardized indirect effect was *CS*_ab_ = − 0.087. Mindfulness explained a larger portion of variance in cognitive reappraisal (27.9%) than in expressive suppression (6.6%) or thought suppression (17.5%). The explained variance for suicidal ideation increased from 11.9 to 13.8%, when cognitive reappraisal was considered as a mediator in the model.

**Table 3:** Total, direct, and indirect effects of the mediation models

	Effect	*SD*	95% CI	*CS*
Mediation model 1 (expressive suppression(M))				
Total effect	− 0.333	0.058	[-0.449, − 0.218]	− 0.345
Direct effect	− 0.291	0.059	[-0.407, − 0.174]	− 0.300
Indirect effect	− 0.044	0.016	[-0.147, − 0.028]	− 0.045
Mediation model 2 (thought suppression(M))				
Total effect	− 0.333	0.058	[-0.449, − 0.218]	− 0.345
Direct effect	− 0.239	0.061	[-0.361, − 0.118]	− 0.248
Indirect effect	− 0.094	0.024	[-0.144, − 0.051]	− 0.098
Mediation model 3 (cognitive reappraisal(M))				
Total effect	− 0.333	0.058	[-0.449, − 0.218]	− 0.345
Direct effect	− 0.250	0.067	[-0.381, − 0.119]	− 0.259
Indirect effect	− 0.084	0.030	[-0.147, − 0.028]	− 0.087

Note. Mediation model 1: trait mindfulness (X), expressive suppression (M), suicidality (Y); Mediation model 2: trait mindfulness (X), thought suppression (M), suicidality (Y); Mediation model 3: trait mindfulness (X), cognitive reappraisal (M), suicidality (Y)

## Discussion

Mindfulness has come to the fore in suicide research as a possible protective factor against suicidal ideation [12]. One construct that is related to both mindfulness [15–18] and suicidal ideation [8, 10, 11] and could, therefore, be a mediating mechanism between these constructs is ERS. The study had two main aims. The first aim was to examine correlations between trait mindfulness and the use of ERS, between trait mindfulness and suicidal ideation as well as suicidal ideation and ERS. The second aim was to examine whether the three ERS mediate the association between trait mindfulness and suicidal ideation.

We hypothesized negative correlations between 1a) trait mindfulness and expressive suppression as well as between 1b) trait mindfulness and thought suppression. We hypothesized 1c) a positive correlation between trait mindfulness and cognitive reappraisal. Hypotheses 1a)-c) could be confirmed. Additionally, we hypothesized a negative correlation between trait mindfulness and suicidal ideation. The second hypotheses could be confirmed. In hypothesis 3, we assumed positive correlation between 3a) suicidal ideation and thought suppression as well as between 3b) suicidal ideation and expressive suppression. We hypothesized 3c) a negative correlation between suicidal ideation and cognitive reappraisal. Hypotheses 3a)-c) could be confirmed. Thus, all correlation analyses revealed the expected results and are in line with earlier studies [16–18]. There was a strong positive correlation between trait mindfulness and cognitive reappraisal and a negative correlation with thought suppression and expressive suppression. The negative correlation between mindfulness and suicidal ideation underlines in addition the findings on MBCT as a method of choice to reduce suicidality [13, 17, 39].

In hypotheses 4 we assumed that all three ERS would show significant mediation effect, which was also confirmed. Results of the first and second mediation model revealed partial mediations of the relationship between mindfulness and suicidal ideation via expressive suppression as well as thought suppression (but only small effects). The last model reveals a partial mediation of cognitive reappraisal on mindfulness and suicidal ideation. In comparison to the other two models, cognitive reappraisal explained the largest amount of variance. When considering that expressive suppression and thought suppression are both unfavorable strategies for emotion regulation, the positive influence of mindfulness on suicidal ideation might be partly explained by the fact that when mindfulness is stronger, dysfunctional emotion regulation strategies are used less often/strongly - presumably in favor of the more frequent use of more functional strategies such as cognitive reappraisal. Accordingly, a lower mindfulness attitude could facilitate the use of unhelpful strategies, such as thought suppression, and thus worsen suicidal symptomatology.

When interpreting the results, the constellation of the sample must be taken into account. It was a relatively young (*M* = 27.35, *SD* = 9.98), mostly female (75%) sample, so findings from the results apply primarily to young women. This makes the results particularly relevant, as it has been shown, that even though deaths from suicide are higher among men than among women, the rate of suicide attempts is significantly higher among women than among men. A meta-analysis [40] also found that research on female-specific risk factors is lacking, which highlight the relevance of the previous study in context of suicide research. In addition, the risk of suicide has increased due to the Covid-19 pandemic, especially for young adults [41, 42]. The results of this study suggest that trait mindfulness influences the choice of ERS. More pronounced trait mindfulness positively influences the choice of cognitive reappraisal for regulating emotions. The cognitive reappraisal strategy might influence and reduce suicidality in accordance with previous research finding that more cognitive reappraisal leads to less suicidality [10, 11]. An explanation for the relationship between mindfulness, cognitive reappraisal, and suicidality discovered here might be that more salient mindfulness attitudes facilitate self-regulation [15] and thereby emotion regulation. One possible explanation is that awareness and acceptance of internal experiences as core elements of mindfulness could make mindfulness a potentially effective factor against avoidance, suppression and excessive focus on negative thoughts [43]. Accordingly, the choice falls rather on an emotion regulation strategy that is considered beneficial, such as cognitive reappraisal. The choice of a beneficial emotion regulation strategy resulting in successfully coping with an emotion leads to greater psychological well-being, which influences suicidality and vice versa.

### Practical implications

The results of this study only indicate that mindfulness influences the ERS but not whether a person is actually able to actively choose one ERS or the other. For clinical practice, our results suggest considering two aspects in treatment especially of young women.

First, a focus of therapy could be on learning strategies to improve ERS. CBT has multiple interventions for actively practicing cognitive reappraisal such as the ABC-model or different disputation techniques for therapists [44]. This might help patients to actively decide for one ERS when there are (especially) negative emotions to cope with. Second, mindfulness interventions could be integrated in therapy of patients at risk for suicidality. This idea is supported by empirical evidence of Mindulness Based Cognitive Therapy (MBCT) as reported for example in a study of Hargus et al. [45] who found in a sample of 27 patients with chronic depression and suicidality that 8 weeks of MBCT reduced depressive symptoms and improved the perception of relapse symptoms for suicidality significantly [45]. Williams et al. [46] substantiated these results in a sample of 274 depressed patients finding MBCT as more effective in relapse prevention than psychoeducation only or treatment as usual. This is especially relevant with regard to the self-reported diagnoses in this specific sample. 53.01% of all participants reported depression as their diagnosis. Therefore, MBCT treatment could be a treatment method for those affected by depression. One explanation why MBCT might be an effective treatment method could be that one typical symptom of depression is the overgeneralization of thoughts. Overgeneralization of thoughts might be reduced on the one hand because mindfulness allows thoughts to pass by without evaluating them and on the other hand allows distancing from those thoughts and perceiving them more as a symptom and not as part of oneself. Complementary, learned strategies for cognitive reappraisal might help patients to change the content of the cognition, which also helps to distance from the negative cognition. It has also been shown that meditation led to an increase in executive control and that these deficits in executive attention as a typical symptom of depression and anxiety orders should be targeted in treatment [21]. Since it has also been shown that mindfulness leads to a higher acceptance of negative life events [47], mindfulness and cognitive reappraisal are important for prevention in any case of psychological distress.

### Strengths

As already stated, the current study is the first to investigate the associations between the three constructs mindfulness, ERS and suicidality. Additionally, trait mindfulness was surveyed, which also represents a gap in the literature. Another strength of the study is the relatively large sample of *N* = 376 people included in the statistical analyses. Even though the sample primarily comprises young women and thus only represents a portion of the entire population, the study provides valuable insight into the suicide risk factors among women, who constitute a majority of suicide attempts [40].

### Limitations and implications for future research

Despite the strengths of the study, there are some limitations that should be considered when interpreting the results. The sample was only recruited online and was both rather young and mostly female. In subsequent research including older and more male subjects, gender and age differences should be examined. Only one item in the survey asked whether the participants were currently suffering from a mental disorder (self-report, no confirmed diagnosis) or had suffered from one in the past. This could be an important confounding factor for the variables surveyed. In future studies, structured clinical interviews could be used to ensure reliable clinical diagnoses. The study was designed cross-sectionally, and retrospective questionnaires were used. This is especially important considering that suicidal ideation is subject to considerable fluctuations over time [48, 49]. There has only been one study examining the daily use of ERS and suicidal ideation. In the frame of a 28-day diary study, Franz et al. [11] found that on a short notice, expressive suppression was helpful to regulate suicidal ideation but on a long term this mechanism was ineffective. They could also show that participants used different ERS depending on their daily form on the respective day. Such so called ecological momentary assessment (EMA) studies allow the repeated assessment of constructs in the natural environment of participants and help to collect more data in their daily life. Considering the temporal instability of the constructs, more EMA studies in this area are needed to identify more factors that might be influenced by trait mindfulness or the other way around that influence suicidal ideation on a short-term notice.

## Conclusions

This study investigated trait mindfulness and its relation to emotion regulation strategies and suicidal ideation. In a cross-sectional online study, correlation analyses revealed that all constructs were correlated emphasizing mindfulness as a potential protective factor that might exert its positive impact on suicidal ideation partly through a heightened use of adaptive and a reduced use of maladaptive emotion regulation strategies. Within this pattern of results, the largest indirect effect of mindfulness on suicidal ideation was found via intensified use of cognitive reappraisal. Our results can inform prevention and therapeutic strategies for suicidal ideation especially for young women.

## Data Availability

All data analyzed in this study are included in the supplementary files.

## Abbreviations

MBCT: Mindfulness Based Cognitive Therapy
ERS: Emotion Regulation Strategy(s)
CBT: Cognitive Behavioral Therapy
